# Urgent need for a non-discriminatory and non-stigmatizing nomenclature for monkeypox virus

**DOI:** 10.1371/journal.pbio.3001769

**Published:** 2022-08-23

**Authors:** Christian Happi, Ifedayo Adetifa, Placide Mbala, Richard Njouom, Emmanuel Nakoune, Anise Happi, Nnaemeka Ndodo, Oyeronke Ayansola, Gerald Mboowa, Trevor Bedford, Richard A. Neher, Cornelius Roemer, Emma Hodcroft, Houriiyah Tegally, Áine O’Toole, Andrew Rambaut, Oliver Pybus, Moritz U. G. Kraemer, Eduan Wilkinson, Joana Isidro, Vítor Borges, Miguel Pinto, João Paulo Gomes, Lucas Freitas, Paola C. Resende, Raphael T. C. Lee, Sebastian Maurer-Stroh, Cheryl Baxter, Richard Lessells, Ahmed E. Ogwell, Yenew Kebede, Sofonias K. Tessema, Tulio de Oliveira

**Affiliations:** 1 African Centre of Excellence for Genomics of Infectious Diseases (ACEGID), Redeemer’s University; Ede, Osun State, Nigeria; 2 Department of Biological Sciences, Faculty of Natural Sciences, Redeemer’s University, Ede, Osun State, Nigeria; 3 Nigeria Centre for Disease Control, Abuja, Nigeria; 4 Institut National de Recherche Biomedicale, Kinshasa, Democratic Republic of the Congo; University of Kinshasa, Kinshasa, Democratic Republic of Congo; 5 Virology Unit, Centre Pasteur of Cameroon, Yaoundé, Cameroon; 6 Institut Pasteur Bangui, Bangui, Central African Republic; 7 Africa Centres for Disease Control and Prevention (Africa CDC), Addis Ababa, Ethiopia; 8 Vaccine and Infectious Disease Division, Fred Hutchinson Cancer Center, Seattle, Washington, United States of America; 9 Howard Hughes Medical Institute, Seattle, Washington, United States of America; 10 Biozentrum, University of Basel, Basel, Switzerland; 11 Swiss Institute of Bioinformatics, Lausanne, Switzerland; 12 Institute of Social and Preventive Medicine, University of Bern, Bern, Switzerland; 13 Geneva Center of Emerging Viral Diseases, HUG, University of Geneva, Geneva, Switzerland; 14 KwaZulu-Natal Research Innovation and Sequencing Platform (KRISP), Nelson R. Mandela School of Medicine, University of KwaZulu-Natal, Durban, South Africa; 15 Centre for Epidemic Response and Innovation (CERI), School of Data Science and Computational Thinking, Stellenbosch University, Stellenbosch, South Africa; 16 Institute of Ecology and Evolution, University of Edinburgh, Edinburgh, United Kingdom; 17 Department of Zoology, University of Oxford, Oxford, United Kingdom; 18 Pandemic Sciences Institute, University of Oxford, Oxford, United Kingdom; 19 Department of Pathobiology and Population Sciences, Royal Veterinary College, London, United Kingdom; 20 Genomics and Bioinformatics Unit, Department of Infectious Diseases, National Institute of Health (INSA), Lisbon, Portugal; 21 Faculty of Veterinary Medicine, Lusófona University, Lisbon, Portugal; 22 GISAID at Laboratorio de vírus respiratórios-IOC/FIOCRUZ, Rio de Janeiro, Brazil; 23 GISAID at Bioinformatics Institute and ID labs A*STAR, Singapore, Singapore; 24 Department of Biological Sciences and YLL School of Medicine, National University of Singapore, Singapore, Singapore; 25 Centre for the AIDS Programme of Research in South Africa (CAPRISA), Durban, South Africa; 26 Department of Global Health, University of Washington, Seattle, Washington, United States of America

## Abstract

The current nomenclature for monkeypox virus is stigmatising and misleading. This Perspective article proposes a practical and neutral system of nomenclature that will allow efficient communication without the risk of further misconceptions, discrimination and stigmatisation.

## Teaser

We propose a novel, non-discriminatory classification of monkeypox virus diversity. Together with the World Health Organization, we named three clades (I, IIa and IIb) in order of detection. Within IIb, the cause of the current global outbreak, we identified multiple lineages (A.1, A.2, A.1.1 and B.1) to support real-time genomic surveillance.

Monkeypox is a disease caused by the monkeypox virus (MPXV) from the Orthopoxvirus genus in the family *Poxviridae* [1,2]. Since the first report of monkeypox virus infection in humans in the 1970s [3], repeated outbreaks have been reported periodically in Western and Central Africa and global events have been detected rarely [4,5]. However, a recent global outbreak of MPVX has been detected without a clear link to Africa [6]. As of 8 June 2022, at least 1111 human cases of MPXV have been confirmed or suspected and cases have been detected in 44 countries [7]. MPXV infection is caused normally by spill-over events to humans from animals such as rodents, squirrels, and non-human primates [1,4,5]. The virus can be also transmitted from one person to another by close contact with lesions, body fluids, respiratory droplets and contaminated materials [1,4]. Case counts and epidemiological patterns suggest that the current global outbreak is sustained by human-to-human transmission [6].

The prevailing perception in the international media and scientific literature is that MPXV is endemic in people in some African countries. However, it is well established that nearly all MPXV outbreaks in Africa prior to the 2022 outbreak, have been the result of spillover from animals to humans and only rarely have there been reports of sustained human-to-human transmissions. In the context of the current global outbreak, continued reference to, and nomenclature of this virus being African is not only inaccurate but is also discriminatory and stigmatizing. The most obvious manifestation of this is the use of photos of African patients to depict the pox lesions in mainstream media in the global north. Recently, Foreign Press Association, Africa issued a statement urging the global media to stop using images of African people to highlight the outbreak in Europe [8].

Although the origin of the new global MPXV outbreak is still unknown, there is growing evidence that the most likely scenario is that cross-continent, cryptic human transmission has been ongoing for longer than previously thought. However, there is a persistent narrative in the media and among many scientists that link the present global outbreak to Africa or West Africa, or Nigeria. Further, the use of geographical labels for strains of MPXV, specifically, references to the 2022 outbreak as belonging to the “West African” or “Western African” clade, strain, or genotype. We therefore believe that a nomenclature that is neutral, non-discriminatory and non-stigmatizing will be more appropriate for the global health community.

## Current classification

In the current classification of MPXV genetic diversity only two clades of MPXV are recognized–referred to as the “West African” clade and the “Central African” or “Congo Basin” clade [9]. However, these historic MPXV clade names are counter to the best practice of avoiding geographic locations in the nomenclature of diseases and disease groups [10,11]. The recent and prompt example implemented for SARS-CoV-2 should be the norm [10]. Given the increasingly rapid communication of, and attention to, the international human MPXV outbreak, it is important to consider an appropriate, non-discriminatory, and non-stigmatizing nomenclature and classification of MPXV clades. In recent publications [12] and symposia, including the WHO Research and Development (R&D) symposium, it was highlighted that the current global outbreak was caused by MPXV of the West African clade. Some genome sequences on the NCBI Genbank database use “West African” for the field “strain” or “genotype”. Like many previous geographic labels of infectious diseases based on locations of first detection, it is misleading and inaccurate because very limited surveillance and limited diagnostic capacity means that the full range of the pathogen is not known. This is crucially demonstrated by the discovery in May 2022 that MPXV has been circulating in over 44 countries without detection and is likely to be present in many more.

## Our proposal: non-discriminatory, non-stigmatizing and neutral classification

Here, we propose a novel classification of MPXV that is non-discriminatory and non-stigmatizing and aligned with best practices in naming of infectious diseases [10] in a way that minimizes unnecessary negative impacts on nations, geographic regions, economies and people and that considers the evolution and spread of the virus.

**Fig 1:**
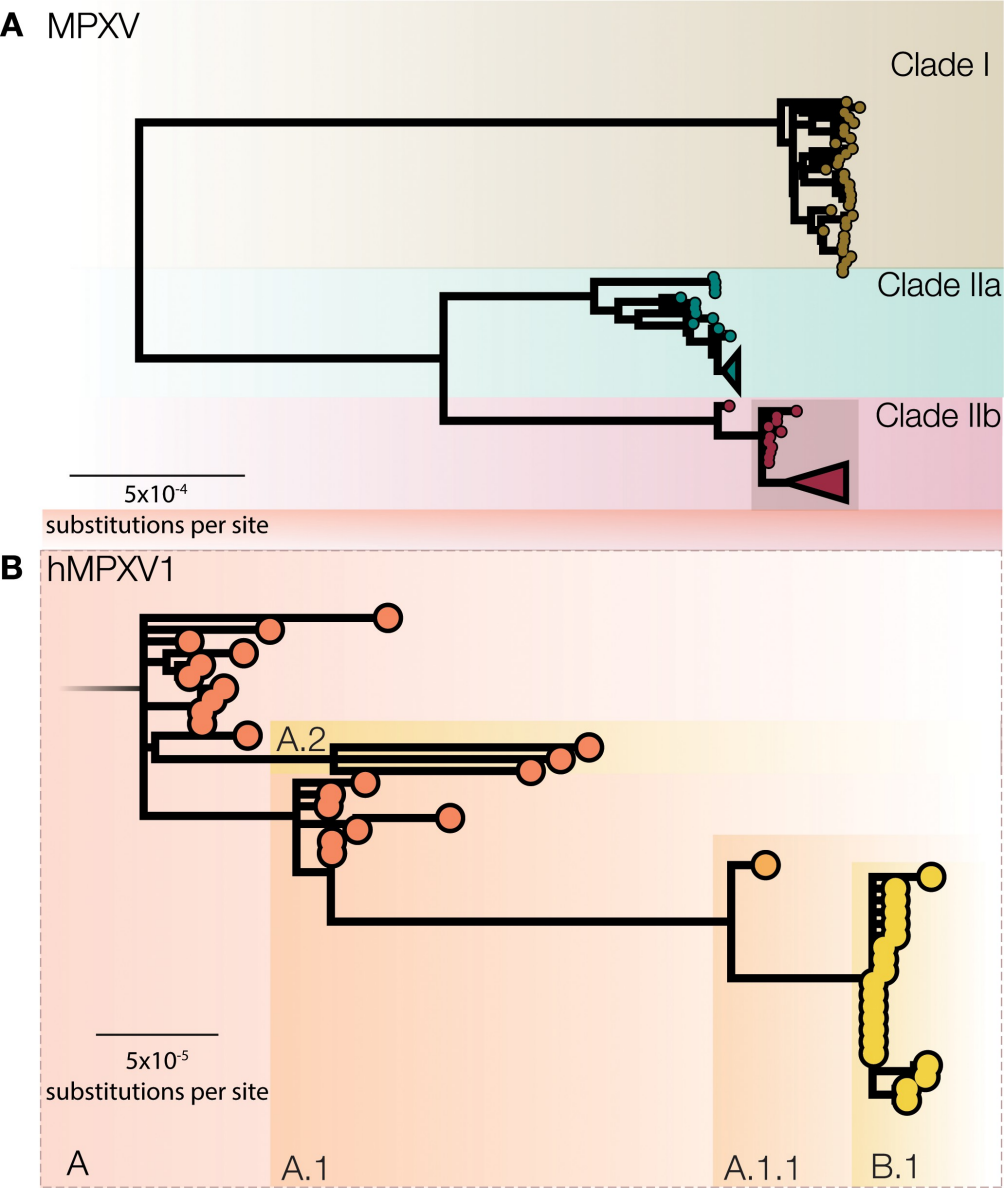
**A)** A midpoint rooted maximum likelihood (iqtree2 using JC model) phylogeny of MPXV genomes sampled from human and non-human infections in 1970–2022 aligned against the reference genome (accession NC_063383) with one of the ITR regions (from 190788 onwards in the genome). A number of repetitive regions were also masked out. Three distinct MPXV clades are indicated, representing the deep diversity of MPXV. Clade I corresponds to the prior “Congo Basin clade”, while Clades IIa and IIb corresponds to the prior “West African clade”. Clade IIb contains a group of genomes from 2017, 2018 and 2022 sampled from human cases that likely represent sustained human-to-human transmission. We propose to label this group hMPXV1. **B)** Proposed nomenclature for genomes belonging to the 2017–2019 outbreaks from the UK, Israel, Nigeria, USA, and Singapore and genomes from 2022 global outbreaks as a fine-scale classification of hMPXV1 virus (MPXV clade IIb) into neutral lineages such as A, A.1, A.1.1, B.1, etc.

In the original proposal, we named MPXV clades 1,2,3 in order of detection, but in discussions with expert committees of the WHO, it was agreed to name these clades I, IIa, IIb to reflect the closer phenotypic and phylogenetic similarity of the two subclades IIa and IIb. These clades include viral genomes from Western African, Central African and localized spillover events in global north countries and from both human and non-human hosts (Fig 1A). Here, clade I corresponds to the prior “Congo Basin clade”, while clades IIa and IIb corresponds to the prior “West African clade”. These three clades represent deep MPXV diversity, accumulated over many years of evolution in the animal reservoir. Further sequencing of MPXV from the animal reservoir may potentially uncover further clades III, IV, V, and so forth.

We also suggest to give the subgroub of clade IIB, containing genomes sampled between 2017–2019 from the UK, Israel, Nigeria, USA, and Singapore and genomes from 2022 global outbreaks, a distinct label (Fig 1B). Since viruses in this group have been transmitting from person to person in dozens of countries and potentially over multiple years, we propose that this represents transmission route distinct from that of previous MPXV cases in humans and should be afforded a distinct name so that it can be referred to specifically in both scientific discourse and the general media. We believe this is an opportunity for a break with the name monkeypox and the historical associations attached to that name. Here we use the placeholder label ‘hMPXV1’ to denote where we believe this now human virus becomes distinct from MPXV [13] (clade IIb; Fig 1B).

Within the hMPXV1 outbreak there is already notable diversity even amongst the limited number of genomes so far described. Thus, we further propose that distinct lineages within the epidemic are given neutral names and suggest a system similar to Pango nomenclature for SARS-CoV-2 [14] with lineages within the hMPXV1 outbreak given labels that encode genealogical relationships. To keep the labels short, we propose to introduce aliases after the second subdivision, instead of after three as in the original Pango scheme. Under this nomenclature, the base of hMPXV1 would be denoted lineage ‘A’, the following clades would be named as ‘A.1’, ‘A.2’, ‘A.1.1’ and the current international 2022 outbreak would be denoted ‘B.1’ as the first detected descendent lineage of ‘A.1.1’ (Fig 1B). A common and regularly updated definition of sequence-based rules to label lineages will aid automated annotation for comparative analysis and scientific discussion in this outbreak. We urge the international community to adopt such a system to preempt the adoption of informal and potentially stigmatizing labels.

With the above suggestions, we encourage the community to adopt a principled and neutral naming scheme for clades and linages such as the one presented here. We believe that this new classification will be easily adopted and is supported by the Africa Centre for Diseases Control and Prevention (Africa CDC) and the WHO [15]. In order to support continuous genomic suveillance, we created an open curator’s group (https://github.com/mpxv-lineages). In this group, we identify new lineages and provide human and machine readable designations for sequence repositories, public health agencies, scientists and the public. Designated lineages can be visualized on Nextstrain (https://nextstrain.org/monkeypox/hmpxv1) and novel sequences can be assigned using Nextclade (https://clades.nextstrain.org). The newly established EpiPox database within GISAID has already adopted this new nomenclature.

We hope that the world uses the current outbreak to advance our understanding, and provides the funding and focus for effective regional and global public health surveillance for emerging and re-emerging threats. By supporting a non-discriminatory and non-stigmatizing classification, we can encourage African and other researchers in low- and middle-income countries (LMICs) to advance genomic surveillance, share sequence data, and minimize negative impacts. Failure to support and adopt the proposed nomenclature and classification may result in loss of interest in sustaining active surveillance and rapid reporting of pathogens with epidemic and pandemic potentials, by scientists and national public health institutions in Africa and other LMICs. Every case of MPXV infection should be treated with the same attention and sense of urgency as the ones now in European countries and North America. The entire epidemic of hMPXV regardless of the location needs to be halted, not just this Northern hemisphere outbreak. A practical and neutral system of nomenclature allows efficient communication without the risk of further misconceptions, discrimination and stigmatisation.
